# Biologic therapy is associated with a mild decrease in the rate of hospitalizations in pediatric IBD

**DOI:** 10.1186/s12887-021-02526-1

**Published:** 2021-02-04

**Authors:** Gil Berkovitch, Shlomi Cohen, Ronit Lubetzky, Dana Singer, Anat Yerushalmy-Feler

**Affiliations:** 1 Pediatric Gastroenterology Unit, Dana-Dwek Children’s Hospital, Tel Aviv Medical Center, 6 Weizmann Street, 6423906 Tel Aviv, Israel; 2 Department of Pediatrics, Dana-Dwek Children’s Hospital, Tel Aviv Sourasky Medical Center, Tel Aviv, Israel; 3 Affiliated to the Sackler Faculty of Medicine, Tel Aviv University, Tel Aviv, Israel

**Keywords:** Children, Hospitalization, Crohn’s disease, Ulcerative colitis, Biologic therapy, Inflammatory bowel disease

## Abstract

**Background:**

The effect of biologic therapy on the incidence of inflammatory bowel disease (IBD)-related hospitalizations is controversial. The high efficacy of biologic agents is weighted against potential therapy-related adverse events, however, there are no data on the effect of biologic therapy on the indications for hospitalization in IBD. We aimed to evaluate the impact of biologic therapy on the indications and rate of hospitalization in pediatric IBD.

**Methods:**

This retrospective cohort study included all children (< 18 years of age) with IBD who were hospitalized in our medical center from January 2004 to December 2019. Data on demographics, disease characteristics and course, and therapy were collected, as were the indications for and course of hospitalizations. We evaluated the relationship between therapy with biologic agents, indications and rates of hospitalization.

**Results:**

Included were 218 hospitalizations of 100 children, of whom 65 (65%) had Crohn’s disease and 35 (35%) had ulcerative colitis. The indications for hospitalization were IBD exacerbations or complications in 194 (89%) and therapy-related adverse events in 24 (11%). The patients of 56 (25.7%) hospitalizations were receiving biologic therapy. In a multivariate analysis, no correlation between therapy and indication for hospitalization was found (*p* = 0.829). Among children under biologic therapy, a decrease in the rate of hospitalizations from 1.09 (0.11–3.33) to 0.27 (0–0.47) per year was observed for patients that were hospitalized during 2016–2019 (*p* = 0.043).

**Conclusion:**

Biologic therapy did not influence the indication for hospitalization, but were associated with a decrease in the rate of hospitalization during 2016–2019 in pediatric IBD.

## Background

Inflammatory bowel disease (IBD) comprises of Crohn’s disease (CD) and ulcerative colitis (UC), which are chronic immune-mediated disorders. IBD is characterized by periods of remissions and exacerbations that often require in-hospital treatment. Several studies have shown a trend of increased IBD-related hospitalizations in both the pediatric and adult IBD populations [1–3]. Vester-Andersen et al. [4] reported that 66% of CD patients and 47% of UC patients had at least one IBD-related hospitalization. Data retrieved from the Swiss IBD cohort showed that almost one-fourth of IBD patients were hospitalized at least once a year [5]. IBD patients may require hospitalizations due to disease exacerbations or complications, therapy-related adverse events, or other comorbidities. Since hospitalizations of IBD patients constitute a heavy burden on patients and families [6], as well as a significant burden on healthcare systems [7–9], modification of the natural history of IBD and reduction in those burdens are major goals of IBD treatment.

Biologic therapy in general and anti-tumor necrosis alpha (TNF-α) agents specifically are an effective therapy for IBD in adults and children [10–14]. The high efficacy of biologic therapy is weighted against potential therapy-related adverse events, primarily a higher risk of infections [15–19]. The effect of those new therapies on hospitalization rate is controversial. Several adult studies have shown that despite their high efficacy, the introduction of biologic agents during the last two decades did not produce significant declines in the rates of IBD-related hospitalizations [20–22]. However, a systematic review and meta-analysis performed by Mao et al. showed that anti TNF-α agents did significantly reduce the hospitalization rate in both CD and UC [23].

Although the incidence of IBD-related hospitalizations has been investigated in depth, data on the effect of biologic therapy on the indications for hospitalizations in IBD are scarce. A focus paper by the epidemiology committee of the European Crohn’s and Colitis Organization identified the obstacles of evaluating this effect, including the difficulty to distinguish between disease-related hospitalizations from those provoked by the adverse events of drug therapy [24]. The increasing use of biologic agents in IBD has raised the question whether a shift toward less exacerbation-related hospitalizations and more adverse event-related hospitalizations might occur. The aim of the current study, therefore, was to evaluate the impact of biologic therapy on the indications and rate of hospitalization in pediatric IBD.

## Methods

### Study population

All children (< 18 years of age) with the diagnosis of IBD who were hospitalized in our medical center from January 2004 to December 2019 were included in this study. The Pediatric Gastroenterology Unit at Dana-Dwek Children’s Hospital in the Tel Aviv Sourasky Medical Center is a tertiary referral center for pediatric patients with IBD. The diagnosis of IBD was made according to the European Society for Pediatric Gastroenterology Hepatology and Nutrition Porto criteria and the revised Porto criteria for the diagnosis of IBD in children and adolescents [25, 26]. We excluded hospitalizations due to conditions that were unrelated to IBD, elective hospitalizations, and hospitalizations due to the first presentation of IBD. Emergency room visits without subsequent hospitalization were not included, nor were re-hospitalizations within one month of a previous hospitalization. IBD-unclassified (IBD-U) patients were included in the UC group for analysis.

### Study design

We retrospectively reviewed the medical records and retrieved the study participants’ demographic data as well as disease characteristics, including IBD location, behavior, and extent according to the PARIS classification [27], IBD course, and therapy. Disease activity was assessed by the pediatric CD activity index (PCDAI) [28] or the pediatric UC activity index (PUCAI) [29]. Disease exacerbation was defined as relapse of clinical symptoms accompanied by elevation < 10 points in the PCDAI or PUCAI. We recorded the course of hospitalization, laboratory and diagnostic work-up findings, and management protocol, and compared the characteristics of hospitalizations due to IBD exacerbations or complications and those due to therapy-related adverse events. Adverse events of therapy were defined as symptoms/diagnoses that are well-known complications of therapy after thorough medical investigation ruled out other recognized etiologies for those symptoms. Pancreatitis was considered a therapy-related rather than a disease-related complication if no IBD exacerbation or small bowel involvement of IBD were identified and after biliary, autoimmune, and traumatic etiologies had been excluded. We specifically evaluated the relation between therapy with biologic agents and indications for hospitalization and the effect of biologic therapy on hospitalization rate.

### Statistical methods

Continuous variables were evaluated for normal distribution by applying histograms and Q-Q plots. Normally distributed continuous variables were expressed as mean and standard deviation (SD). Non-normally distributed continuous variables were expressed as median and interquartile range (IQR). Categorical variables were presented as frequency and percentage. The categorical variables were compared between indication for hospitalization by means of the chi-square test or Fisher-exact test, and continuous variables were compared with the independent samples t-test or Mann-Whitney test logistic regression was applied for the multivariate analysis with the backward stepwise method (the Wald test was used as criteria, and *p* > 0.1 was taken as the threshold for removal). Wilcoxon signed rank test was employed to compare the number of hospitalizations per year before and after treatment with biologic agents. All of the statistical tests were 2-tailed. A *p* value of < 0.05 was considered significant. SPSS was used for all statistical analyses (IBM Corp. Released 2016. IBM SPSS Statistics for Windows, Version 24.0. Armonk, NY: IBM Corp.)

The study was approved by the Institutional Review Board of the Tel-Aviv Medical Center (TLV-0266-18).

## Results

### Patient population

**Fig. 1:**
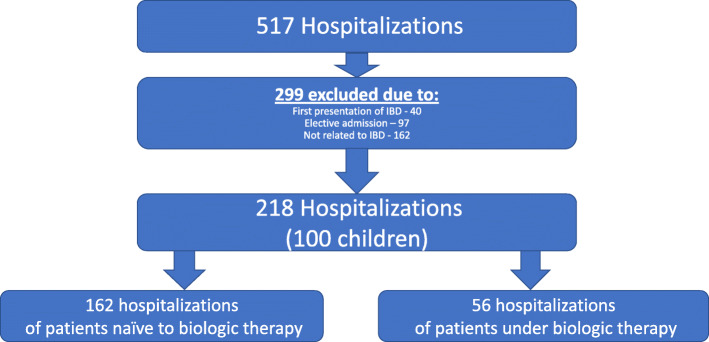
Hospitalization flowchart

Overall, 517 hospitalizations were recognized. Of them, 299 were excluded: 40 hospitalizations were due to the first presentation of IBD, 97 were elective admissions and 162 hospitalizations that were unrelated to IBD (Fig. 1). We included 218 hospitalizations of 100 children, 65 (65%) with CD and 35 (35%) with UC. Of an overall pediatric IBD cohort consists of 280 patients in our center (179 CD patients and 101 UC or IBD-undetermined patients), these 100 children were hospitalized at least once – a rate of 36.3% in CD and 34.7% in UC. Their demographic and clinical data are presented in Table 1. The number of hospitalizations of the patients was as follows: 51 (51%) had one hospitalization, 19 (19%) had two, 16 (16%) had three, and 14 (14%) had 4–10 hospitalizations.

**Table 1:** Demographic and clinical data of the study cohort

Variable	All *n* = 100	Crohn’s disease *n* = 65	Ulcerative colitis *n* = 35	*p*
Age at diagnosis (years)	12.2 (9.28–14.3)	12.2 (9.5–14.35)	12 (6.3–14.2)	0.522
Males, *n*	49 (49%)	33 (50.8%)	16 (45.7%)	0.630
Disease location				
Ileo-cecal (L1)		22 (33.8%)		
Colonic (L2)		18 (27.7%)		
Ileo-colonic (L3)		25 (38.5%)		
Upper GI tract (L4)		8 (12.3%)		
Disease behavior				
Inflammatory (B1)		55 (84.6%)		
Stricturing (B2)		5 (7.7%)		
Penetrating (B3)		5 (7.7%)		
Perianal involvement		8 (12.3%)		
Disease extent				
Proctitis (E1)			3 (8.6%)	
Left-sided colitis (E2)			4 (11.4%)	
Extensive colitis (E3)			2 (5.7%)	
Pancolitis (E4)			26 (74.3%)	
Disease severity				
Never severe (S0)			22 (62.9%)	
Ever severe (S1)			13 (37.1%)	
Extra-intestinal manifestations	21 (21%)	16 (24.6%)	5 (14.3%)	0.226
BMI Z-score	−0.4 [(−1.4)-(+ 0.3)]	−0.8 [(−2.1)-(+ 0.1)]	0.05 [(− 0.6)-(+ 0.83)]	0.142

*GI* gastrointestinal, *BMI* body mass index

The indications for hospitalization included 194 (89%) IBD exacerbations or complications and 24 (11%) therapy-related adverse events. The therapy-related complications included 10 (41.7%) infectious conditions (4 skin, 4 pneumonia, 1 chickenpox, and 1 urinary tract), 8 cases of pancreatitis (33.3%), and one case each of drug-induced hepatitis, drug reaction with eosinophilia and systemic symptoms, acute tubular necrosis, vomiting, fever of unknown origin, and Hodgkin lymphoma.

### Characteristics of hospitalizations by indication

The characteristics of hospitalizations due to disease exacerbations and therapy-related adverse events are described in Table 2. There were no differences in age, gender, disease type, length of hospitalization, or work-up between the two indications for hospitalizations.

**Table 2:** Characteristics of hospitalizations due to IBD exacerbation and those due to therapy-related adverse events

Characteristic	Hospitalizations due to IBD exacerbations *n* = 194	Hospitalizations due to therapy-related adverse events *n* = 24	***p***
Age (year)	14.9 (11.6–16.5)	16.2 (13.3–17.1)	0.277
Males, *n*	93 (47.9%)	9 (37.5%)	0.439
Crohn’s disease	131 (67.5%)	16 (66.7%)	0.944
Ulcerative colitis	63 (32.5%)	8 (33.3%)	
Duration from IBD diagnosis (year)	2.4 (0.7–4.3)	1.6 (0.4–4.2)	0.801
Therapy at admission			
5-ASA	101 (52.1%)	17 (70.8%)	0.082
Corticosteroids	40 (20.6%)	6 (25%)	0.619
Enteral nutrition	40 (20.6%)	4 (16.7%)	0.649
Methotrexate	5 (2.6%)	0	> 0.999
Azathioprine	69 (35.6%)	13 (54.2%)	0.116
Biologic agents	49 (25.3%)	7 (29.2%)	0.679
Disease activity at admission			< 0.001
Remission	0	7 (29.2%)	
Mild	104 (47.5%)	13 (54.2%)	
Moderate	85 (38.8%)	4 (16.6%)	
Severe	30 (13.7%)	0	
Length of hospitalization	4 (2–6.3)	6 (3–9)	0.934
Blood cultures	88 (45.4%)	14 (58.3%)	0.299
Stool culture	54 (27.8%)	6 (25%)	0.746
Imaging studies	129 (66.5%)	19 (79.2%)	0.243
US	69 (35.6%)	13 (54.2%)	0.125
CT	22 (11.3%)	1 (4.2%)	0.312
MRI	3 (1.5%)	0	> 0.999
Endoscopy			
Upper GI endoscopy	17 (8.7%)	2 (8.4%)	0.942
Lower GI endoscopy	26 (13.4%)	1 (4.2%)	0.229
Antibiotics	98 (50.5%)	11 (45.8%)	0.709
Blood transfusion	13 (6.7%)	1 (4.2%)	0.601
Surgery	14 (7.2%)	0	0.374
Abscess drainage	10 (5.2%)		
Ileo-cecectomy	1 (0.5%)		
Others	3 (1.5%)		

*IBD* inflammatory bowel disease, *5-ASA* 5-aminosalicylic acid, *US* ultrasound, *CT* computed tomography, *MRI* magnetic resonance imaging, *GI* gastrointestinal

### Correlation between therapy with biologic agents and indication for hospitalization

The patients in 162 (74.3%) hospitalizations were naïve to biologic therapy. The 21 patients in 56 (25.7%) hospitalizations were receiving biologic therapy: 40 (71.4%) infliximab, 10 (17.9%) adalimumab, 5 (8.9%) vedolizumab, and 1 (1.8%) golimumab. The hospitalizations of patients under biologic therapy included 16 (28.6%) who were treated with combination therapy (14 azathioprine and 2 methotrexate). In addition to biologic agents, in 24 hospitalizations (42.9%) the patients were treated with 5-ASA compounds.

Most of the hospitalizations of the biologic naïve patients (*n* = 144, 88.9%) were due to IBD exacerbation and 18 (11.1%) were due to therapy-related adverse events. Fifty (89.3%) hospitalizations of patients undergoing biologic therapy were due to IBD exacerbation and 6 (10.7%) were due to therapy-related adverse events (*p* = 0.945). The characteristics of hospitalizations in patients under biologic therapy compared to biologic-naïve patients are presented in Table 3.

**Table 3:** Characteristics of hospitalizations in patients under biologic therapy compared to biologic-naïve patients

Characteristic	Hospitalizations of patients under biologic therapy *n* = 56	Hospitalizations of patients naïve to biologic therapy *n* = 162	***p***
Age (year)	15.4 (12.2–17.2)	14.8 (11.3–16.4)	0.124
Males, *n*	29 (47.9%)	73 (45.1%)	0.385
Crohn’s disease	31 (55.4%)	116 (71.6%)	0.025
Ulcerative colitis	25 (44.6%)	46 (28.4%)	
Duration from IBD diagnosis (year)	3.15 (1.14–4.13)	2 (0.5–4.15)	0.070
Indication of hospitalization:			0.945
IBD exacerbation	50 (89.3%)	144 (88.9%)	
Therapy-related adverse events	6 (10.7%)	18 (11.1%)	
Therapy at admission			
5-ASA	24 (42.9%)	94 (58%)	0.049
Corticosteroids	9 (16.1%)	37 (22.8%)	0.285
Enteral nutrition	14 (25%)	30 (18.5%)	0.297
Methotrexate	2 (3.6%)	3 (1.9%)	0.459
Azathioprine	14 (25%)	68 (42%)	0.024
Disease activity at admission			0.755
Remission	3 (5.3%)	4 (2.5%)	
Mild	23 (41.1%)	69 (42.6%)	
Moderate	23 (41.1%)	66 (40.7%)	
Severe	7 (12.5%)	23 (14.2%)	
Length of hospitalization	5 (2.75–7)	4 (2–7)	0.136
Blood cultures	32 (57.1%)	70 (43.2%)	0.072
Stool culture	19 (33.9%)	41 (25.3%)	0.213
Imaging studies	35 (62.5%)	113 (69.8%)	0.316
US	16 (28.6%)	66 (40.1%)	0.105
CT	5 (8.9%)	18 (11.1%)	0.647
MRI	1 (1.8%)	2 (1.2%)	0.760
Endoscopy			
Upper GI endoscopy	3 (5.3%)	16 (9.9%)	0.301
Lower GI endoscopy	6 (10.7%)	21 (13%)	0.660
Antibiotics	30 (53.6%)	79 (48.8%)	0.385
Blood transfusion	6 (10.7%)	8 (4.9%)	0.129
Surgery	3 (5.4%)	11 (6.8%)	0.706
Abscess drainage	2 (3.6%)	8 (4.9%)	
Ileo-cecectomy	0	1 (0.6%)	
Others	1 (1.8%)	2 (1.2%)	

*IBD* inflammatory bowel disease, *5-ASA* 5-aminosalicylic acid, *US* ultrasound, *CT* computed tomography, *MRI* magnetic resonance imaging, *GI* gastrointestinal

A multivariate analysis adjusted for age, gender, and disease type failed to show any correlation between therapy and indication for hospitalization (*p* = 0.829). In addition, no correlation between therapy and indication for hospitalization was found in a sub-analysis of CD and UC separately.

Of the cohort, 65 (29.8%) hospitalizations were during 2004–2011 and 153 (70.2%) hospitalizations were during 2012–2019. In the former period, 51 of the biologic naïve patients (*n* = 54, 94.4%) were due to IBD exacerbation while 10 hospitalizations of patients undergoing biologic therapy (*n* = 11, 90.9%) were due to IBD exacerbation (*p* = 0.656). In the latter period, 94 of the biologic naïve patients (*n* = 108, 87%) were due to IBD exacerbation while 39 hospitalizations of patients undergoing biologic therapy (*n* = 45, 86.7%) were due to IBD exacerbation (*p* = 0.951).

### Hospitalization rates before and after biologic therapy

Overall, 34 children (34%) were treated with biologic agents before or after hospitalization and had a median follow-up of 5.35 (2.93–7.63) years. Sixteen children (47.1%) received infliximab, 8 (23.5%) infliximab and adalimumab, 5 (14.7%) adalimumab, 2 (5.9%) infliximab and vedolizumab, 2 (5.9%) vedolizumab, and 1 (2.9%) infliximab and golimumab. The median number of hospitalizations per year in these children was 0.47 (0–2.13) before biologic therapy compared to 0.33 (0–0.85) after biologic therapy, representing a decrease of 0.24 [(− 0.37)-(+ 2.08)] hospitalization per year (*p* = 0.086). The rate of hospitalizations in patients under biologic therapy was not affected by disease behavior (*p* = 0.968 for inflammatory phenotype and *p* = 0.568 for penetrating or stricturing CD).

Of these 34 children, 8 had a first hospitalization during 2004–2011 and 26 were first hospitalized during 2012–2019. In the former period, the median number of hospitalizations per year was 0.19 (0–0.61) before biologic therapy compared to 0.33 (0.28–0.63) after biologic therapy (*p* = 0.271). In the latter period, the median number of hospitalizations per year was 0.72 (0.14–3.12) before biologic therapy compared to 0.33 (0–0.92) after biologic therapy (*p* = 0.081). Of 16 patients that were hospitalized during 2016–2019, the median number of hospitalizations per year was 1.09 (0.11–3.33) before biologic therapy compared to 0.27 (0–0.47) after biologic therapy (*p* = 0.043).

## Discussion

The effects of biologic therapy on the incidence of IBD-related hospitalizations are controversial, and currently available data on those effects in pediatric IBD are scarce. This study included 218 hospitalizations of 100 pediatric IBD patients, and the results showed that 89% of the hospitalizations were due to IBD exacerbations or complications, while 11% were due to therapy-related adverse events. The use of biologic agents did not change the indication for hospitalization. Moreover, there was no change in the rate of hospitalization before and after treatment among the children receiving biologic therapy, except for patients that were treated during 2016–2019. In concurrence with the reported increase in the incidence of pediatric IBD [30], the rate of biologic therapy and, specifically, anti-TNF-α agents, is increasing worldwide among both adults and children [31–34] and is now the cornerstone in the treatment of IBD. This trend caused a significant increase in healthcare costs attributable to IBD [35–37]. Importantly, hospitalizations represent major events in the natural history of IBD and an important marker of severity for predicting future outcome [23].

Since biologic therapy was claimed to achieve better disease control compared to traditional IBD therapies along with some potential serious adverse events, it is noteworthy that most of IBD-related hospitalizations in our study were due to IBD exacerbations or complications while the minority were due to therapy-related adverse events. Importantly, we did not observe any relation between therapy with biologics and indication for hospitalization. The lack of a shift toward more hospitalizations resulting from adverse events of biologic agents could be explained by a lower rate of adverse events in real-life compared to the number of adverse events recorded during clinical trials, or, alternatively, frequent but mild adverse events that do not require hospitalization. Moreover, the pediatric population with fewer comorbidities may be at lower risk of therapy-related adverse events compared to the adult population. Furthermore, while a difference between characteristics of hospitalizations due to IBD exacerbation and those due to therapy-related complications could be anticipated, all hospitalizations in our study were similar in terms of patient age, gender, disease type, length of hospitalization, and work-up evaluation.

The impact of the introduction of biologic therapy on disease-related hospitalizations has been investigated in previous studies with inconsistent results. Comparable to the current study that showed a similar hospitalization rate before and after biologic therapy, several studies, mainly in the adult population, reported no change in the rates of IBD-related hospitalizations [20, 38]. Other studies observed an increase in hospitalizations in the last two decades and an increased risk of hospitalization in patients treated with thiopurines, corticosteroids, and anti-TNF-α agents [22, 39]. In contrast, Ballou et al. [40] showed that although the frequency of IBD emergency department visits was increasing, the rates of hospitalizations decreased. Ehteshami-Afshar et al. [41] further showed that the relative risk of hospitalizations in IBD patients treated with infliximab was 0.61. Anti-TNF-α agents significantly reduced hospitalization (OR = 0.46 in CD and OR = 0.48 in UC) compared to placebo in a systematic review that included a meta-analysis [23]. Additionally, Costa et al.’s systematic review and meta-analysis [42] concluded that infliximab reduced hospitalizations in patients with IBD, and Targownik et al. [35] recently observed a decline in CD-attributable hospitalization costs. Importantly, we showed that although biologic therapy had no effect on the indication of hospitalization, patients that were hospitalized during 2016–2019 had a lower rate of hospitalizations after the initiation of biologic therapy. This may reflect the changes in standard of care in biologic therapy IBD, for example therapeutic drug monitoring, the treat to target approach, “top down” versus “step up” etc.

The effect of biologic agents on the rate of IBD-related surgery is also a controversial matter. Several studies reported reductions in surgery rates among IBD patients treated by biologic agents [42–44], while others reported no change [20, 22, 34]. It is important to note that the use of biologic agents in clinical practice involves a greater variability in patient selection, dosing, timing, monitoring, and optimization of therapy. A better optimization of the use of biologic agents and a more personalized approach may improve patient morbidity and health outcomes.

In parallel with a reduced risk of developing IBD complications under biologic therapy [45], adverse events of therapy are worth noting. Immunosuppression and infections are among the most significant adverse events of biologic therapy in IBD. However, recent studies proposed that the risk was not as high as had originally been claimed. Lichtenstein et al. [46] reported that infliximab treatment did not affect the incidence of infection, mortality, or malignancy in adults with IBD, and no evidence of increased risk of serious infections in IBD patients under immunosuppression was found by Wheat et al. [47]. The systematic review and meta-analysis of Bonovas et al. [48] concluded that biologic agents increase the risk of opportunistic infections in patients with IBD, but not the risk of serious infections. These claims that therapy-related adverse events are not a significant indication for hospitalizations in adult IBD patients support our comparable findings on pediatric IBD patients.

To the best of our knowledge, this is the first study to describe indications of hospitalization in pediatric IBD in the era of biologic therapy. The major strength of our study lies in the data having been based on a large demographic and clinical database of children with IBD [49], that enabled us to specifically analyze patients receiving biologic therapy. The study is limited by its retrospective nature and by the challenge to correctly differentiate between disease exacerbation and therapy-related adverse events. The fact that hospitalization is an outcome that may be affected by time period, provider, local admission criteria and other factors may limit the applicability of our results to other centers. The patients were treated with other medications that are also potentially associated with adverse events. However, most of the patients were treated with biologic agents as monotherapy, and the correlation between biologic agents and the indication for hospitalization was performed while controlling for other IBD-related therapies. The wide range of a 16-year follow-up of the cohort in which there had been major advancements in the therapeutic paradigm is another potential limitation of the study, although this wide range increases the power of the study to detect potential changes in the pattern of hospitalizations and sub-analysis to shorter time-periods was performed. The lack of a significant change in the pattern and rate of hospitalizations under biologic therapy may be affected by the small sample size and by an inherent bias of initiating biologic therapy in patients with more severe disease that may need more frequent medical care. Nevertheless, we tried to overcome this limitation by performing the comparison of hospitalization rates only for patients that were treated with biologic therapy, before and after the initiation of therapy.

## Conclusions

In conclusion, IBD exacerbations continue to be the major indications for hospitalizations of pediatric IBD patients. Our current findings suggest that biologic therapy did not influence either the indication or the rate of hospitalizations, except a small group of patients that were treated during 2016–2019. A better optimization of the use of biologic agents may have the potential to improve health outcome, including the hospitalization rate and the course of disease. Future controlled prospective studies are needed to confirm these conclusions. New therapeutic options for improving disease control may also reduce the risk of hospitalization in pediatric IBD patients.

## Data Availability

The datasets used and/or analyzed during the current study are available from the corresponding author on reasonable request for clinical research purposes.

## Abbreviations

IBD: Inflammatory bowel disease
CD: Crohn’s disease
UC: Ulcerative colitis
TNF- α: Anti tumor necrosis alpha
IBD-U: IBD-unclassified
PCDAI: Pediatric CD activity index
PUCAI: Pediatric UC activity index
SD: Standard deviation
IQR: Interquartile range
